# Transcranial Direct Current Stimulation to Ameliorate Post-Stroke Cognitive Impairment

**DOI:** 10.3390/brainsci14060614

**Published:** 2024-06-19

**Authors:** Kelly L. Sloane, Roy H. Hamilton

**Affiliations:** 1 Department of Neurology, Perelman School of Medicine, University of Pennsylvania, Philadelphia, PA 19104, USA; 2 Department of Physical Medicine and Rehabilitation, Perelman School of Medicine, University of Pennsylvania, Philadelphia, PA 19104, USA

**Keywords:** cognition, neurorehabilitation, transcranial direct current stimulation, non-invasive bran stimulation, aphasia, neglect, neuroplasticity

## Abstract

Post-stroke cognitive impairment is a common and disabling condition with few effective therapeutic options. After stroke, neural reorganization and other neuroplastic processes occur in response to ischemic injury, which can result in clinical improvement through spontaneous recovery. Neuromodulation through transcranial direct current stimulation (tDCS) is a promising intervention to augment underlying neuroplasticity in order to improve cognitive function. This form of neuromodulation leverages mechanisms of neuroplasticity post-stroke to optimize neural reorganization and improve function. In this review, we summarize the current state of cognitive neurorehabilitation post-stroke, the practical features of tDCS, its uses in stroke-related cognitive impairment across cognitive domains, and special considerations for the use of tDCS in the post-stroke patient population.

## 1. Introduction

With a prevalence of almost 800,000 annually in the United States, stroke is the leading cause of adult disability [1]. Estimates of the prevalence of cognitive dysfunction after stroke vary considerably depending on the type of cognitive testing and the timing after stroke, but estimates range from 30% to up to 90% [2,3]. Older adults and those with vascular risk factors are at a 3.6-fold increase in the risk of poststroke dementia [4]. Every 1-year increase in baseline age results in 17% higher odds of cognitive impairment at 1 year post-stroke [5]. Mild or subtle cognitive dysfunction can sometimes be missed early in the post-stroke period. Presence of post-stroke cognitive impairment, no matter the degree of impairment, is associated with poorer functional outcomes as well as increased caregiver burden, healthcare costs, and mortality [6,7,8]. Even among patients with good overall functional outcomes, cognitive impairment persists in over 70% of cases after 3 months [9]. Attention and memory are among the most common stroke-related deficits [3,10] with a negative impact on daily functioning and responsiveness to rehabilitation [11,12,13,14]. Despite how common and disabling post-stroke cognitive impairment can be, there are few effective therapies. In this review, we describe a promising intervention, transcranial direct current stimulation (tDCS), to treat post-stroke cognitive impairment by leveraging neuroplastic processes.

The gold standard for treatment of post-stroke cognitive impairment remains one-on-one or group cognitive therapy sessions with a licensed rehabilitation specialist. Rehabilitation strategies for specific cognitive functions, such as training of memory, have been used with the aid of external memory aides, visual scanning training, and problem-solving techniques [15]. The success of cognitive and speech therapies varies depending on insurance status, socio-economic factors, and accessibility, and time-intensive and prolonged courses of therapy are often not offered beyond the immediate post-acute recovery period [16]. Pharmacological therapies like donepezil and other anticholinesterase inhibitors, which are commonly used in the treatment of Alzheimer’s disease, have shown limited benefit in post-stroke cognitive impairment [17]. Physical activity, particularly aerobic exercise, has been associated with a reduced risk of dementia in older adults [18] and similarly in individuals with stroke-related cognitive impairment; the therapeutic effects of exercise have been identified, but the durability of these effects are not yet known [19,20]. Similarly, cognitive rehabilitation techniques have demonstrated improvement in cognitive function in the short-term post-treatment period, but the long-term effects are not known [21,22]. Other treatments, like mindfulness-based stress reduction, have also been tested in small pilot studies with short-term benefits [21].

Many treatment strategies are derived from approaches to other neurological conditions affecting cognition, like neurodegeneration or cognitive aging. However, they do not leverage post-stroke neuroplastic processes occurring in the immediate post-stroke period. Forms of non-invasive neuromodulation have the potential to modulate the spontaneous recovery already occurring in neural networks. As such, non-invasive brain stimulation has emerged as a promising intervention to treat cognitive impairment in stroke survivors [23]. One such approach, tDCS, is a promising, low-cost, accessible technique that has been demonstrated to be an effective tool for improving cognitive dysfunction in healthy adults as well as those with a variety of brain pathologies [24,25,26,27]. However, for the stroke patient population, the majority of neuromodulation studies have focused on motor rather than cognitive impairments.

In this review, we will provide an overview of the application of tDCS as an intervention for augmenting cognitive recovery after stroke. We will first discuss the current state of cognitive rehabilitation in post-stroke recovery. Next, we will describe the mechanisms of action of tDCS and the utility of tDCS to augment cognitive performance in non-stroke patient populations. We will then review the existing studies of tDCS to improve cognitive performance in post-stroke cognitive impairment and discuss future applications.

## 2. Current State of Cognitive Neurorehabilitation in Post-Stroke Recovery

### 2.1. Cognitive Profile

Cognition is an umbrella term for specific higher-order functions and can be divided into at least six neurocognitive domains: language, learning and memory, social cognition, complex attention, executive function, and perceptual-motor function [28]. Cognitive dysfunction and dementia frequently occur following an acute stroke, and they are an important cause of stroke-related morbidity [29]. Approximately 66% of patients show cognitive impairment in the acute phase [30], and 50% of stroke survivors fulfil criteria for mild cognitive impairment after 18 months [31,32]. In addition to impairments in global cognition, deficits in attention and executive functions are common [33]. Despite the prevalence of post-stroke cognitive impairment, most research in stroke recovery focuses on non-cognitive impairments. In fact, a recent review found that only 4.6% of peer-reviewed research publications involving stroke survivors included a measurement of cognition [34].

Estimates of the incidence and prevalence of cognitive dysfunction after stroke vary. Some studies suggest that vascular cognitive impairments affect up to 90% of people after stroke [3,9]. Other studies give lower estimates at 10–35%, likely because of differences in assessments or definitions of dementia in hospital and population-based studies [35]. In addition to age and ischemic small vessel white matter disease, which are known mediators of post-stroke cognitive impairment [36], neuroimaging findings associated with post-stroke cognitive impairment include: microbleeds, brain atrophy, medial temporal atrophy, and lesions in strategic brain locations (e.g., right corticospinal tract, left arcuate fasciculus, left middle frontal gyrus, and left postero-inferior cerebellum) [37,38].

Cognitive deficits are associated with worse outcomes. Individuals who are cognitively impaired 3 months after stroke have a 53% increased risk of death at 5, 10, and 15 years after stroke compared to those without cognitive impairment [6]. Among patients with motor deficits in the arm after stroke, those with attention and executive function difficulties demonstrated worse recovery than those with intact cognition [39]. Further, cognitive dysfunction after stroke, as defined by the Montreal Cognitive Assessment (MoCA) of less than 26, was associated with increased functional impairment with a modified Rankin score (mRS) greater than 2 at 6, 12, and 36 months after stroke [7]. Even among patients who show “good” functional outcomes, as defined by mRS as less than 2, more than half have persistent cognitive dysfunction, most commonly affecting memory, visuospatial, and executive functioning [3,40]. In addition to causing new post-stroke cognitive impairment, stroke can also accelerate pre-existing cognitive decline due to underlying neurodegenerative disease [2].

### 2.2. Impact of Cognitive Impairment on Other Modalities of Recovery

There is a growing body of evidence that post-stroke cognitive impairment is associated with worse recovery and less responsiveness to therapies for language and motor impairments [40,41,42] and functional dependence [43]. Learning in any modality post-stroke engages cognitive processes [44]. Motor performance may be affected by cognition function, especially involving tasks with increasing cognitive demands [39,45]. In a study of subacute stroke patients at 3 months after stroke, impairments in motor function were associated with impairments in memory and executive function [46]. Worse cognitive function post-stroke was associated with lesser responsiveness to a gait intervention [47] and completion of complex motor tasks [48]. The full extent of how cognitive status modulates response to rehabilitation interventions may not be fully understood, as many studies of post-stroke motor impairment limit participation based on cognitive status (i.e., cognitive impairment is an exclusion for participation) [39].

### 2.3. Interventions to Improve Post-Stroke Cognitive Impairment

Cognitive rehabilitation provided by speech-language pathologists or occupational therapists is the current gold standard of care for cognitive recovery. High-intensity rehabilitative training can alleviate impairments in cognitive functions [21,49], with greater than 50 min per day of cognitive therapy associated with improved cognitive outcomes, compared to lesser duration of therapy [50]. However, in real-world practice, patients participate in much less therapy than required for successful recovery [51,52]. Clinical factors, such as stroke severity and discharge destination, and non-clinical factors, such as insurance status, socio-economic factors, and accessibility, can limit opportunities for rehabilitative training [53,54,55]. In addition, even when available, the therapies themselves require substantial effort, and these motivational demands are often perceived as overwhelming and exhausting [56].

Other interventions to improve cognition after stroke have been studied with limited success. Randomized controlled trials of pharmacological interventions, such as donepezil [57], escitalopram [58], and amantadine [59], have revealed small but significant improvements in cognitive performance. Virtual reality [60], computer training [61], and aerobic exercise [62] have also shown promise.

## 3. tDCS Mechanism, Considerations and Other Uses

### 3.1. Mechanism of Action

tDCS is a non-invasive form of brain stimulation that can target specific brain regions integral to the cognitive network. tDCS uses constant, low direct current through external electrodes, which penetrate the skin, skull, and meninges to modify neural transmembrane electrical potentials and, therefore, the excitability and firing rate of neurons [63]. In normal neuronal function, the neuronal resting membrane potential is influenced by the movement of ions across the cell membrane. Depolarization refers to the increased positive membrane potential, and conversely, hyperpolarization refers to the negative membrane potential. To generate an action potential, the opening of the voltage-gated sodium channels leads to the influx of positive sodium ions into the cell such that the membrane potential becomes more positive, and depolarization occurs. In tDCS, a low intensity electrical current can alter the resting membrane potential in the neurons near the electrode [64]. Neurons in proximity to the anode exhibit increased depolarization of their transmembrane potential, and neurons underlying a cathode appear to exhibit hyperpolarization of their transmembrane potential [65,66]. The cortical effects of anodal and cathodal tDCS are often conceived of as being excitatory and inhibitory, respectively; however, the relationship between stimulation polarity and elicited cortical effects can be more complicated, depending on parameters such as stimulation intensity and duration [67]. Alterations in transmembrane potentials appear to last up to an hour, cause a local effect, and are N-methyl-D-aspartate (NMDA) receptor dependent [68]. Magnetic resonance imaging (MRI) studies have demonstrated increased perfusion and functional connectivity during stimulation [69]. The administration of tDCS has also been found to be very safe, with adverse effects that include fatigue, mild headache, nausea, and itching in the area of stimulation [70].

### 3.2. Practical Features of tDCS

**Figure 1:**
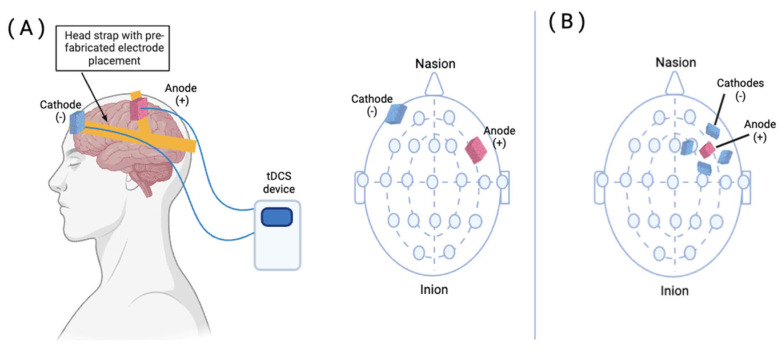
Transcranial direct current stimulation (tDCS) setup including battery operated device, electrodes (cathode and anode), head strap on which electrodes are placed. Electrode placement is based on 10–20 electroencephalogram (EEG) montage. Panel (**A**) is conventional tDCS set up where the cathode is placed on the right supraorbital region and the anode is placed on the left dorsolateral prefrontal cortex, a montage commonly used for treatment of post-stroke cognitive impairment. Panel (**B**) is a high definition tDCS in a 4 × 1 set up where 4 cathodes surround the anode, which is placed in the left dorsolateral prefrontal cortex. Created with BioRender.com.

The practical features of tDCS make it an especially useful tool for neurorehabilitation. Devices are small and portable, primarily comprising a battery-powered device, two electrode wires (anode and cathode), and a head strap. This device delivers a unidirectional, constant current, with the anode and cathode placed in different locations on the scalp, depending on the functional outcome desired. Once a stimulation target is selected, the corresponding location can be found using 10–20 electroencephalogram (EEG) system measurements. While this approach is less precise anatomically, it does not require neuronavigation technologies or physiologic localization using transcranial magnetic stimulation [71]. Several options exist for the positioning of electrodes on the head in the target location. Some device models employ adjustable rubber straps to accommodate each individual person’s head size, while others use pre-fabricated headgear, which can be used for a range of head sizes and has electrode sites at pre-determined montage locations. The components of a conventional tDCS device are illustrated in Figure 1. Many tDCS devices can be employed with relatively little training, and self-administration of tDCS or delivery by a caregiver is being explored with several devices.

Guidelines for technology and protocol implementation have been developed to direct the remote use of tDCS in clinical research [72]. Remote-use devices, which are designed be to compact and user friendly, can be pre-programmed with stimulation parameters such that the participant cannot alter the stimulation dose, duration, or frequency [72]. These parameters can be controlled through hardware or software limitations that prevent indiscriminate device use. With respect to hardware-based limitations, a device is pre-programmed to provide a set number of sessions (e.g., 5 sessions of 20 min each) with defined intensity (e.g., 2 milliamps anodal stimulation) within a set interval (e.g., 1 session per 24 h interval). Regarding software-based limitations, a device is programmed to provide stimulation at a specific intensity, duration, and session frequency and can only be activated with a unique code. Effective and safe remote tDCS administration requires not only these safeguards on device administration but also operationalization of remotely supervised tDCS, including comprehensive protocols for training participants in the use of tDCS and ongoing monitoring for compliance, treatment-associated adverse effects, and the user’s capability to self-administer tDCS. Capacity for remote use invites a broader and more accessible intervention, but it also risks inconsistencies in use when it is self-administered by the participant rather than the research staff. These challenges may be mitigated by requiring hands-on pre-intervention training for research participants to learn and practice with the technology and real-time monitoring of treatment fidelity through videoconferencing [72,73,74,75,76]. A single center reported findings from its studies involving 6779 participants who underwent remotely supervised tDCS and found no serious adverse events, few study discontinuations due to tolerability (0.04%), and there was high treatment fidelity (92–98%) [75]. Remote tDCS has been used in studies of a wide variety of neuropsychiatric conditions including multiple sclerosis [72], Parkinson’s disease [74], major depressive disorder [76], depression, [77], and stroke [78], to name a few.

Similar to conventional tDCS, wherein stimulation is performed through a single pair of large (often 5 × 7 cm or 5 × 5 cm) electrodes, high-definition tDCS (HD-tDCS) has been developed to deliver a focused current to the target site. HD-tDCS employs multiple smaller electrodes to focus the current in one or more regions of the brain and has substantially higher spatial resolution than conventional tDCS. For example, a common HD-tDCS montage is the 4 × 1 ring, in which a center electrode is placed over the target cortical region surrounded by four return electrodes [79].

### 3.3. Models of Neuroplasticity to Guide Stimulation Targets

**Figure 2:**
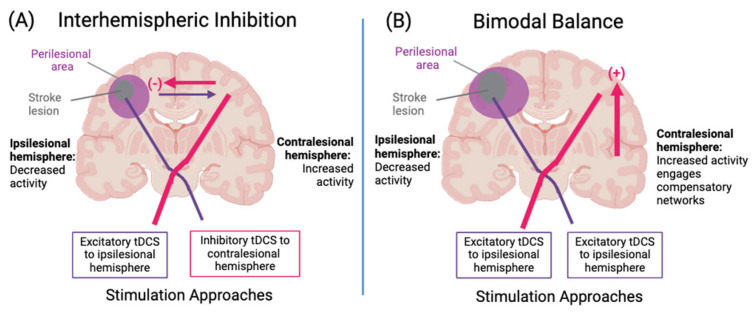
Models of stroke recovery. Panel (**A**) illustrates the Interhemispheric Inhibition model, in which perilesional areas increase activity to compensate for the stroke lesion. The transcallosal inhibitory influence from the contralesional hemisphere leads to an imbalanced inhibitory force on the ipsilesional hemisphere. Under this model, excitatory stimulation is applied to the ipsilesional hemisphere or inhibitory stimulation is applied to the contralesional hemisphere. Panel (**B**) illustrates the Bimodal Balance model, in which contralesional hemisphere structures form a compensatory reserve network resulting in enhanced cortical excitability of the contralesional hemisphere with the effects (beneficial or detrimental to recovery) dependent on the degree of structural reserve. Under this model, excitatory stimulation is applied to both hemispheres. Created with BioRender.com.

The anatomic targets of tDCS are typically at the level of the cortex, as it is much more difficult to reliably direct current to subcortical or brainstem sites. Additionally, stimulation targets are typically brain regions that are hypothesized directly related to the impairment being targeted. There are two central models that have historically guided how brain regions are selected as targets for stimulation (Figure 2). The first model—the interhemispheric imbalance model—is based on the notion that functional impairments are mediated, at least in part, by a mismatch in brain activity between cerebral hemispheres induced by unilateral injury of the brain. This model is predicated on the notion that many of the connections between the two cerebral hemispheres are inhibitory in nature and that intact cerebral hemispheres, therefore, normally exert an inhibitory influence on each other. According to this account, a unilateral injury to the brain results in (1) decreased cortical excitability on the injured (i.e., ipsilesional) hemisphere, (2) decreased inhibitory projections to the intact (i.e., contralesional) hemisphere, and (3) further inhibition of the ipsilesional hemisphere mediated by increased contralesional interhemispheric inhibition [80]. This implies that two intervention strategies for improving the imbalance between ipsilesional and contralesional hemispheres would be to either increase the excitability of the ipsilesional hemisphere or decrease the activity of the contralesional hemisphere. This version of the interhemispheric imbalance model has motivated a number of studies, employing tDCS and other forms of noninvasive neuromodulation in motor recovery [81,82], neglect [83], and aphasia [83,84,85,86,87].

In the second model—the bimodal balance recovery model—the residual structural brain reserve influences physiology and functional output [88]. For individuals with low structural reserve (i.e., greater stroke-induced structural brain injury) especially, increased cortical excitability of the contralesional side will provide compensatory function and activity in residual brain networks [88]. These models have been developed from the perspective of motor recovery, where motor output is largely attributed to the corticospinal tract, but there is not consensus on an optimal region for stimulation in cognitive rehabilitation. Functional connectivity studies have demonstrated that cognitive operations are organized along interconnected networks rather than represented in discrete processing modules [89]. In certain forms of cognitive impairment like aphasia or neglect, where discrete hubs of activity have been identified, the site of stimulation is predominantly the left inferior frontal gyrus [86] and the right posterior parietal cortex [83], respectively. However, for domain-general cognitive functions, any hub within the network of interest could serve as a potential stimulation site. The dorsolateral prefrontal cortex (DLPFC) has emerged as a popular site of stimulation. The DLPFC is a crucial hub for multiple cognitive networks (dorsal attention network, salience network, frontoparietal network) that support processing and response involved in executive function, working memory, motor planning, and attention [90,91,92] and is a common target for stimulation because of its role in these cognitive domains [93].

### 3.4. Task-Dependency of tDCS and Approaches to Therapy

The effect of tDCS depends on the selection of the site of stimulation and the concurrent training task. The task-dependency relates to the fact that tDCS is believed to induce subthreshold changes in the neuronal resting membrane thresholds [94,95]. However, neuroplastic changes associated with tDCS are thought to be induced via Hebbian principles of neuronal activation (i.e., “neurons that fire together wire together”) and are dependent on the presence of recurrent, organized firing of relevant neuronal pathways [96,97,98]. By this account, the mechanistic goal of tDCS is to modulate existing patterns of neuronal firing in order to further strengthen or weaken those firing patterns. For this reason, it has been argued that stimulation must be paired with training tasks that activate the brain regions or networks that are being stimulated [99]. For example, the ipsilesional primary motor cortex would be an appropriate target for the amelioration of stroke-induced hemiparesis, and constraint-induced motor therapy of that hemiparetic arm would be an appropriate training task because it is designed to engage the ipsilesional motor cortex through forced use of the paretic limb.

### 3.5. Brain State-Dependency for tDCS and Priming

The brain state of the individual prior to stimulation may also play an important role in response to subsequent tDCS and its effects [100]. Thus, priming involves optimizing the pre-stimulation brain state to achieve greater plasticity-related changes during the stimulation period [101]. Priming has been evaluated through several methods, including physical exercise, cognitive exercises, or stimulation itself. Aerobic exercise has been shown to improve cognitive outcomes in older adults with cognitive impairment, [102] and aerobic exercise is also associated with increased cortical excitability and decreased intracortical inhibition, conditions favorable for plasticity [103]. Thus, aerobic exercise’s synergistic effects may prime the brain for focal modulation [104], which is currently being studied with an ongoing clinical trial [105]. Emotional stress may also be a priming method for enhancing the effects of tDCS. Acute stress activates stress-related neural circuits, including the DLPFC, through the release of cortisol and catecholamines to coactivate the targeted network [106,107] and, therefore, alter the neural system’s responsiveness to stimulation [106]. Studies of healthy adults have indicated that stress priming is associated with better performance on working memory tasks, particularly those involving emotional content [108]. In addition, tDCS itself can be used to prime the brain resting state to improve responsiveness to tDCS. Facilitatory tDCS protocols have been developed with subthreshold, low-intensity cathodal stimulation subthreshold, low-intensity priming sessions using cathodal tDCS to elicit low-level prior neuronal activity, enhancing the likelihood of further long-term potentiation (LTP)-like effects without significantly impacting overall excitability [109,110,111].

### 3.6. Limitations of tDCS

There are several key limitations of tDCS in its applications for cognitive enhancement. First, the low current of tDCS leads to a subthreshold alteration of the resting membrane potential and supports neuron depolarization but does not directly alter action potential and depolarization [112]. Therefore, the effect of tDCS is less robust than other neuromodulatory modalities like transcranial magnetic stimulation (TMS). Second, tDCS has limited spatial accuracy due to its non-invasive administration over the scalp in approximate locations. Computational models of current flow in conventional tDCS show diffuse spread over cortical regions [113]. Techniques like high-definition tDCS can constrict current to specific regions through multiple smaller electrodes to improve spatial resolution. Third, although tDCS has been studied in a number of neurologic and psychiatric conditions, the heterogeneity of study designs have made it difficult to determine the optimal stimulation parameters and treatment protocols, especially for post-stroke cognitive impairment [114].

### 3.7. Comparison of tDCS to Other Neuromodulatory Techniques Used in Post-Stroke Cognitive Impairment

Several other neuromodulatory techniques exist that can also be considered for intervention in post-stroke cognitive impairment. Similar to tDCS, transcranial magnetic stimulation (TMS) involves passing an electric current through conductive wires of an insulated coil to induce a local magnetic field, which then stimulates a secondary electric current in the brain underlying the coil. Repetitive TMS (rTMS), a therapeutic form of TMS, uses high frequency (at or above approximately 5 pulses per second) stimulation, which can induce the promotion of synaptogenesis and long-term potentiation [115]. Because of the mechanism of action of TMS, induction of seizures is a possible severe acute adverse effect. Though relatively rare, seizures have been described in single-pulse, pattern, and repetitive pulse uses of TMS [116,117,118]. In practice, researchers are advised to avoid individuals with risk factors for TMS-provoked seizures, which include mediations or medical conditions known to lower seizure threshold and history of epilepsy [119]. While tDCS can be administered remotely, which increases accessibility, rTMS is hindered by its size and expense. The use of the device is limited to trained practitioners rather than individuals for administration. Similar to tDCS, TMS lacks good spatial resolution, which can diffuse the cortical effects of the stimulation [120,121]. The effect of rTMS has been investigated in a number of post-stroke cognitive impairment cases, most commonly motor impairment and language. In the assessment of global cognitive function, as measured by the Mini-Mental Status Examination (MMSE) or the Montreal Cognitive Assessment (MoCA), active rTMS targeting the left DLPFC was associated with improved performance compared to sham [122], with evidence for positive effects in post-stroke language and motor impairments as well [123].

Another form of neuromodulation, transcranial focused ultrasound (TUS), delivers highly focused acoustic energy at low frequency [124]. TUS has the advantage of greater spatial resolution than other forms of non-invasive brain stimulation [125,126]. Transcranial focused ultrasound can open the blood–brain barrier [127] and when applied in short bursts, can modulate cortical neuron excitability [128]. The study of transcranial ultrasound stimulation has largely focused on its applications of clot dissolution and delivery of therapeutic drugs to specific brain regions [129], but it is a promising intervention to ameliorate post-stroke cognitive impairments needing further research.

## 4. tDCS Therapy in Stroke Patient Populations

The effect of tDCS on cognition has been studied in several non-stroke patient populations, including those with normal cognition, neurodegenerative disease, and traumatic brain injury [130,131]. In healthy adults, tDCS can improve performance in the learned tasks [132] and result in enhanced functional connectivity of working memory networks on fMRI evaluation [133]. In those with mild cognitive impairment or Alzheimer’s disease (AD), tDCS seems to have an enhanced effect in participants with mild cognitive impairment or AD compared to healthy controls [134]. In demyelinating disorders, like multiple sclerosis, neuromodulation has also shown benefits in cognitive performance and fatigue when paired with cognitive training for multiple sclerosis patients [135,136]. Additionally, when evaluated in participants with cognitive and behavioral changes secondary to traumatic brain injury, tDCS with stimulation to the DLPFC resulted in improvements in overall cognition as well as psychosocial wellbeing [137,138].

### 4.1. tDCS for Cognitive Performance in Stroke: Language

Methods of tDCS use to improve cognitive performance have varied in the stroke patient population based on the domain targeted. Aphasia, an impairment in language production and comprehension, can be caused by injury to the language-dominant hemisphere of the brain, most commonly the left. Studies of tDCS to improve language production in persons with chronic left hemisphere stroke and non-fluent aphasia have revealed positive effects associated with stimulation [139,140,141,142]. Though functional MRI data suggest that recovery is associated with restoration of the left hemispheric residual language network [143], other studies suggest that language recovery relies on an intact right hemisphere [144,145]. Therefore, there is no consensus on the optimal target for tDCS for aphasia-related impairments. The majority of clinical trials have focused on excitatory stimulation to the left hemispheric sites of language function, like the left inferior frontal gyrus or the left posterior perisylvian region, to equilibrate the interhemispheric inhibition proposed in the previously described model. To date, the largest randomized, double-blind, sham-controlled trial in this area included 74 participants with chronic non-fluent post-stroke aphasia with the stimulation target determined by pre-treatment functional MRI, in which the anode was directed over the area of greatest activation in the left hemisphere during spoken naming over the course of 15 language therapy sessions. Active tDCS resulted in a greater change in correct picture naming and in the correct naming relative to sham, with the benefit maintained for at least 3 weeks [146]. Another smaller study investigated the long-term outcome of tDCS to the left frontal (perilesional) region while participating in simultaneous naming training for 10 sessions, resulting in improvements in naming that persisted until 16 weeks post-intervention [147]. Anodal stimulation over the left inferior frontal gyrus, a site that mediates language control corresponding to Broca’s area, combined with repetition tasks, has been used in over 19 studies of post-stroke aphasia and resulted in improved accuracy in speech production and naming [148,149], speech production [150], and reading ability [151,152].

Though the left hemisphere is the most common stimulation side, other regions like the right hemisphere and the cerebellum have also been investigated for treatment of post-stroke aphasia. The effect of stimulation of the opposite hemisphere, the contralesional right hemisphere, builds on the bimodal balance model, in which cathodal (inhibitory) stimulation targets the source of compensatory activity in the contralesional hemisphere. In a randomized controlled crossover study, 10 individuals with naming impairments due to post-stroke aphasia underwent 5 days of 2 mA cathodal stimulation over the right Broca’s homologue area or sham stimulation for 20 min with simultaneous daily sessions of conventional word-retrieval training [152]. Cathodal stimulation resulted in improved naming on the Boston Naming Test post-intervention (after the final session), but durability of this result beyond the immediate period was note assessed. A meta-analysis of cathodal tDCS to the right hemisphere for post-stroke aphasia found three studies, including Kang et al. above, and pooled analysis showed no statistically significant difference between cathodal stimulation and sham stimulation [153]. Another potential site of stimulation is the right cerebellum because of its role in word retrieval and generation, somatic processing, and language learning [154]. The cerebellum, while involved in language pathways, remains intact in individuals with isolated left hemisphere lesions, therefore representing an intact gateway to the affected neural networks [155]. Hillis et al. have investigated anodal stimulation to the right posterolateral cerebellum in combination with computerized aphasia treatment in a double-blinded, within-subject crossover randomized controlled trial, in which participants undergo sham stimulation along with either anodal or cathodal (2 mA) stimulation for 15 sessions of 20 min each [156]. Active stimulation resulted in greater improvements in naming accuracy compared to sham immediately post-intervention and at 2 months post-intervention, with the cathodal stimulation showing larger (though not statistically significant) gains than anodal stimulation. The mechanism of this polarity-independent effect is not clear but may be the result of the complexity of gyral folding in the cerebellar cortex such that the structure orientation relative to the electric field direction may differ even within small regions of the cerebellum [157].

### 4.2. tDCS for Cognitive Performance in Stroke: Attention

In addition to aphasia, the largest body of work in post-stroke cognitive impairment and tDCS focuses on a common form of inattention, neglect. Neglect, the inability to perceive, report, and orient to sensory events towards one side of space, is typically caused by injury to the non-dominant (right) hemisphere in the parietal cortex of the brain, resulting in impairments affecting the left side. Though damage to several areas of this hemisphere can produce neglect, cortical regions of the right inferior parietal lobe or temporo-parietal junction have classically been implicated in neglect [158]. Recovery studies involving tDCS to ameliorate neglect have used the right posterior parietal cortex (PPC) as the primary target for anodal stimulation, likely because of its role in visuospatial attention, sustained attention, and spatial perception [159]. A recent systematic review found 11 studies (152 participants with stroke-related impairments) with the tDCS intervention ranging from 1 to 20 sessions and intensity ranging from 1 to 2 mA [83]. Out of the 11 studies, 9 applied anodal stimulation to the ipsilesional right PPC, with 5 of those applying cathodal stimulation to the contralesional PPC. Two studies stimulated the right primary motor cortex. All studies reported improvements in measures of neglect, including line bisection and cancellation tests in the active stimulation groups compared to sham stimulation; however, the strength and generalizability of these findings are unclear given the heterogenous study designs.

Several studies in neglect-related impairments have involved dual stimulation, in which the anodal is placed on the ispilesional PPC and the cathode is placed on the contralesional hemisphere to disrupt the inhibitory imbalance that occurs during spontaneous recovery [160,161]. A double-blind random-order cross-over experiment investigated dual tDCS in chronic stroke participants, with spatial neglect evaluated in three randomly arranged tDCS sessions of 1 mA for 20 min (anodal tDCS over the right PPC and cathodal tDCS over the left PPC, anodal tDCS over the right PPC and cathode over the left supraorbital area, and sham mode). Significant changes in line bisection test and star cancelation test performance were noted in both stimulation conditions compared to sham, with dual tDCS having a stronger effect than single tDCS [162]. A similar study tested tDCS in three similar modes (anodal tDCS over the right PPC, cathodal tDCS over the left PPC, or sham tDCS) at 2 mA for 30 min over 15 sessions, but dual stimulation was not applied. Though each group experienced improvements in the line bisection test, the star cancellation test, and the motor-free visual perception test, there was no statistically significant difference among groups [160]. A double-blinded, pilot randomized clinical trial enrolled participants with subacute stroke (within 6 months of enrollment) of 1 mA for 20 min for a total of 15 sessions in three treatment conditions: anodal tDCS over the right PPC, cathodal tDCS over the left PPC, or sham tDCS. Post-tDCS session, participants received 1 h of physical therapy involving visual scanning and task-specific activities. Anodal tDCS was associated with better performance on tests of neglect than sham, but there were no statistically significant differences between anodal and cathodal stimulation [161].

The primary motor cortex is another stimulation site that has been investigated in neuromodulation of neglect. Because the primary motor cortex mediates early consolidation of motor memories and movement dynamics [163], anodal stimulation of this region may facilitate the realignment of hand-eye coordination leftward to consolidate learning from during prism adaptation, a form of behavioral therapy [164].

### 4.3. tDCS for Cognitive Performance in Stroke: Other Domains of Cognition

#### 4.3.1. Global Cognition

Unlike the consistency of stimulation targets and outcome measures in neuromodulatory studies of neglect and aphasia, studies of neuromodulation to target other domains of cognition are varied. Because cognitive function is conveyed through diffuse, interconnected, bilateral networks, there are no clear stimulation targets; however, several have been tested [165,166]. A recent study of tDCS included 26 patients with chronic stroke and post-stroke cognitive impairment who underwent 20 sessions of either tDCS plus computerized cognitive training or sham tDCS plus computerized cognitive training. Participants were trained to self-administer (or a caregiver administered) tDCS for 5 sessions per week for 4 weeks, with each session lasting 30 min. Participants undergoing active stimulation of the DLPFC showed significant improvements in the Korean version of the MoCA (*p* = 0.004 for real group versus *p* = 0.132 for sham group), more notable in individuals with lower pre-intervention MoCA scores and those with left hemispheric lesions [78].

A recent meta-analysis found 11 randomized controlled trials published in English and Chinese journals, all occurring within the last 10 years [23]. Overall, treatment with anodal tDCS was more likely to result in improved cognitive performance (*p* < 0.001) at the end of follow-up. Improvement appears to be more pronounced when stimulation was administered < 40 days from incident stroke.

#### 4.3.2. Memory and Verbal Learning

Neuroimaging studies have identified regions of the prefrontal and parietal cortices as key regions for verbal learning and working memory [167]. One trial of 10 participants with chronic stroke stimulated the left dorsolateral prefrontal cortex for 2 sessions; and compared to sham stimulation, the tDCS group showed significant changes in working memory recognition accuracy and response time [168]. A similar study included 11 patients with chronic stroke who underwent stimulation 5 times per week during their inpatient rehabilitation hospitalization while participating in computer-assisted cognitive exercises. Anodal stimulation was to the bilateral prefrontal cortex and the cathode to the non-dominant arm for 30 min at an intensity of 2 mA; the intervention group demonstrating significant improvements in auditory and visual continuous performance tests compared to sham [169]. Another study involved anodal or sham tDCS to the left temporoparietal area to enhance audiovisual memory in 12 stroke patients. There was marked improvement in the number of correctly recalled words across 5 trials on Rey Auditory Verbal Learning in the tDCS group compared to sham [165]. In Yun et al. [166], the anterior temporal lobe, a key hub for semantic memory [170], was the focus of stimulation. Forty-five participants with a history of stroke were randomized to anodal stimulation of the left anterior temporal lobe, the right anterior temporal lobe, or sham stimulation with intervention delivered for 30 min for 15 sessions. There was greater improvement seen in the left anodal stimulation group compared to the two other groups across multiple cognitive assessments but only a statistically significant difference in the verbal learning test-delayed recall (*p* < 0.05) [131].

#### 4.3.3. Executive Function

Executive function, a group of cognitive processes related to self-regulation, planning, and reasoning for goal-directed behavior [22], is thought to be regulated by the frontal (particularly DLPFC) [171] and the temporal lobes [172]. As with working-memory-related impairments, tDCS for executive-function-related impairments have included several studies targeting the left DLPFC. A meta-analysis of 27 studies that targeted the DLPFC found substantial heterogeneity in elements of the study design: duration (10–30 min), training task (e.g., N-back, Flanker task, Affective Flexibility, etc.), outcome measures, intensity (1–2 mA), and timing (during or after stimulation) [173]. In pooled analyses, anodal, cathodal, or bilateral tDCS did not yield a significant effect on general executive function. However, in evaluation of subdomains of executive function, there was a significant effect of anodal tDCS on updating tasks but not inhibiting or shifting. Other tDCS studies focused on executive function have also targeted the superior temporal gyrus. In Hosseinzadeh et al. [174], 100 participants with chronic ischemic stroke were divided into 4 groups (anodal tDCS to the left superior temporal gyrus, cathodal tDCS to the left superior temporal gyrus, and anode at the contralateral supraorbital region, sham, and control), where participants underwent 3 sessions of 30 min per week for 1 month. The group undergoing anodal tDCS to the left superior temporal gyrus showed significant increases in the Trail Making Test, a measure of executive function, after 1 and 3 months post-stimulation when compared to the three other groups, suggesting durability of the intervention effect.

#### 4.3.4. Perceptual Motor Function

Perceptual motor function involves the acquisition of a motor skill without the awareness of learning using the interpretation of sensory information [175]. Perceptual disorders, though they affect almost 75% of stroke survivors [176], often go undetected. Though many studies have focused on motor strength improvement with tDCS, few studies have focused specifically on perceptual motor function and tDCS, but several have stimulated the ipsilesional primary motor cortex paired with motor exercises of the affected limb [177]. Anodal tDCS may enhance induction for consolidation, but its effects on long-term retention are still unknown [178].

#### 4.3.5. Social Cognition

Social cognition, a domain of cognition, refers to one’s ability to process, judge, and apply information about social behaviors, including that of oneself and others [179], and is mediated by the right temporal parietal junction (TPJ) [180]. Studies of tDCS in stroke patient populations have not focused on amelioration of social cognition, but studies from healthy controls may provide insight into the potential effects in individuals with post-stroke cognitive impairment. One study of healthy controls evaluated anodal, cathodal, or sham tDCS to the right TPJ at 1 mA for 20 min [181]. Anodal stimulation was associated with enhanced imitation control and visual perspective taking, which are components of social cognition, but did not affect attribution of mental states. Conversely, cathodal stimulation of the right TPJ with 1.5 mA current for 20 min was associated with decreased cognitive empathy, further supporting evidence that cortical excitability in this region may be an important stimulation target for impairments in social cognition among individuals with stroke and other acquired brain injuries [182].

### 4.4. Considerations Unique to Post-Stroke Patient Populations

#### 4.4.1. Medications Acting on the Central Nervous System

Based on the mechanism of action of tDCS, medications acting on the central nervous system can alter the effects of stimulation, including ones commonly used or prescribed during the care of stroke patients, like nicotine, amphetamines, and selective serotonin reuptake inhibitors (SSRIs). Nicotine administration in non-smokers was found to dampen the excitability typically enhanced under anodal tDCS and diminish the excitability, reducing aftereffects under the cathodal tDCS [183,184]. Though the mechanism is not clearly defined, based on animal models, nicotine modulates nicotinic acetylcholine receptors to elicit neuronal depolarization at presynaptic sites, thus modulating glutamate and dopamine release [185,186]. SSRIs are commonly prescribed in individuals with stroke-related hemiparesis to promote motor recovery or to treat post-stroke depression or pain [187]. With anodal stimulation, citalopram can amplify and prolong tDCS-induced excitability after even a single dose, with the effect thought to be NMDA-receptor dependent [188,189]. A similar medication class, selective norepinephrine reuptake inhibitor (SNRI), has also demonstrated enhanced excitability with anodal tDCS [190]. Chronic use of both SSRIs and SNRIs with cathodal stimulation converts the expected long-term depression-like plasticity into facilitation [188,190]. Serotonin can impact the influx of calcium ions through NMDA receptors to enhance neuronal depolarization [191]. Similar effects are seen in amphetamines, which is an NMDA receptor antagonist [192]. Medications blocking dopamine receptors reduced both the typical anodal excitability enhancement and the cathodal excitability reduction [193]. Conversely, administration of a D2 agonist has been shown to eliminate the aftereffects of tDCS under both the anode and cathode [194]. D2/D3 agonists, such as ropinirole, have been demonstrated to reverse the anodal aftereffects of tDCS at low dosages but have no effect with medium doses; at high dosages, ropinirole decreased the magnitude of anodal stimulation aftereffects [193].

#### 4.4.2. Chronicity of Intervention

After an ischemic stroke, destruction of neural networks stimulates network reorganization following injury [195]. The mechanisms involve the activation of cell repair and genesis and the unmasking of pre-existing subthreshold connections or the creation of new connections [196,197]. In cognitive networks, the interactions are interhemispheric, and post-stroke dynamic functional connectivity was significantly increased in the bilateral prefrontal cortices immediately after stroke [198]. Indeed, synaptic connectivity related to complex functions like memory or cognition are distributed throughout the cortex [199]. Human and animal studies have revealed a period of heightened neuroplasticity, often referred to as a “sensitive period”, that ends in the first month after stroke in rodent models [200] and in the first 3 months after stroke in humans [201,202]. During this time, dynamic molecular, electrophysiologic, and structural reorganization occurs [196,201]. Cortical circuit remapping in the peri-infarct region is activity dependent aided by underlying synaptic plasticity, which ensures neurons receive adequate synaptic input [203] and redistribute synaptic strength through neural circuits [196,204]. These changes on a neural level are also reflected clinically; the most spontaneous recovery after stroke is also observed clinically during this sensitive period [205,206].

Thus, neuromodulation through tDCS may augment spontaneous neuroplastic processes occurring during the subacute period after stroke. Further, post-stroke rehabilitation studies indicate that delayed initiation of therapies are associated with worse functional outcomes and longer hospital length of stays [207]. However, the majority of stroke recovery studies, including those involving neuromodulation, involve intervention in the chronic stage, where spontaneous recovery is thought to be static. A recent meta-analysis found that the effects of tDCS were more pronounced when performed <40 days after stroke compared to 40 days or more after stroke: Χ^2^ = 5.11 and *p* = 0.02 [23]. There were only 4 studies involving a total of 233 participants that evaluated tDCS in this early subacute period, and there have been no studies directly comparing neuronal effects of tDCS across different timepoints after stroke. Our knowledge of the underlying neuroplastic processes occurring during the first 3 months post-injury suggests that the timing for optimal impact of an intervention, especially one like tDCS that modulates plasticity, may be in the early subacute period.

#### 4.4.3. Neurovascular Effects

In addition to its effect on synaptic plasticity, tDCS can modulate changes in cerebral perfusion. Both anodal and cathodal stimulation increase cerebral blood flow with the magnitude in healthy adults [208]. In animal models of stroke, cathodal tDCS can restore perfusion in ischemic tissue at risk of infarction through several mechanisms, including collateral perfusion enhancement by inducing vasodilation [209]. Direct vascular response to electrical current originates from the activation of perivascular nerves evoked by electrical current and a robust neurovascular response [210]. In studies involving humans, cerebrovascular vasomotor reactivity, the vasodilatory capacity of cerebral vasculature in response to internal molecular signals, was reduced with anodal stimulation and amplified with cathodal stimulation [211]. Pilot studies of tDCS in acute ischemic stroke have shown that cathodal stimulation is associated with increased cerebral blood volume and perfusion through induction of vasodilation [212]. In addition, cathodal stimulation is also postulated to provide direct neuroprotection by inhibiting peri-lesional excitotoxic and inflammatory effects [209]. These findings—cathodal stimulation can have a neuroprotectant effect through the reduction in excitotoxic responses and through increased cerebral perfusion—may appear to contradict the interhemispheric inhibition and bimodal balances models, which would suggest cathodal stimulation of the lesioned region would be detrimental. These models account for neuroplasticity-related changes that occur in the post-acute period after stroke but not the immediate post-injury period, in which a lack of perfusion (e.g., vessel occlusion) causes ischemic injury. The perfusional benefits of cathodal stimulation may provide benefits in the hyperacute period by restoring cerebral blood flow to hypoperfused areas and reducing the apoptotic and inflammatory cascades during neuronal cell death. As such, tDCS may be a promising direction for acute stroke management to alleviate hypoperfused ischemic regions.

## 5. Conclusions

Though the consistency of study designs has been variable, tDCS appears to be a promising adjunct to cognitive therapy. Patients typically receive the most intensive cognitive therapy during the acute to subacute stages after stroke, and based on previous research on cognitive recovery, the greatest improvement from post-stroke cognitive impairment occurs within the first 6 months [213]. Therefore, it is reasonable that the effects of tDCS would be enriched if delivered during this time period. In the stroke patient population, published studies of tDCS for cognitive recovery are limited by small sample sizes and inconsistent study design, and randomized controlled trials are needed to assess efficacy.

The body of literature currently available is limited by small study samples, heterogenous study designs, and limited long-term follow up. Further research is indicated to determine several key factors to optimize tDCS as an intervention for post-stroke cognitive impairment. Larger, well-designed trials are needed to assess not only the granular effects of tDCS (i.e., improvement on an individual cognitive task) but also generalizability to other functional outcomes. In addition, stimulation parameters need to be identified in order to standardize intervention protocols, which will facilitate pooled analyses across studies. This method of intervention may be personalized based on clinical characteristics and neuroimaging factors, but further research is needed to determine which variables predict responsiveness to tDCS. And equally importantly, research is needed to establish if neuromodulation combined with cognitive therapy affects functional outcomes in ways which are meaningful to individual circumstances and needs, central to the concept of patient-centered care; the implementation of a therapy that could speed the rate of recovery of cognitive skills and may also impact quality of life for individuals with stroke.

## Data Availability

No new data were created or analyzed in this study. Data sharing is not applicable to this article.
